# Cellular Immune Response in Patients Immunized with Three Vaccine Doses of Different Vaccination Schemes Authorized by the Chilean Ministry of Health in January 2022

**DOI:** 10.3390/life12040534

**Published:** 2022-04-05

**Authors:** Paz Beatriz Tabilo Valenzuela, Gabriela Flores Balter, Gustavo Saint-Pierre Contreras, Daniel Conei Valencia, Catalina Moreno Calderón, Constanza Bohle Venegas, Marcia Guajardo Rivera, Francisco Silva Ojeda, Maria Jesus Vial Covarrubias

**Affiliations:** 1 Servicio de Laboratorio Clínico, Hospital Clínico Universidad de Chile, Santiago 8380000, Chile; gabriela.flores@ug.uchile.cl (G.F.B.); gsaintpierre@hcuch.cl (G.S.-P.C.); cmorenoc@hcuch.cl (C.M.C.); cbohle@hcuch.cl (C.B.V.); mguajardo@hcuch.cl (M.G.R.); fsilva@hcuch.cl (F.S.O.); mjesus@hcuch.cl (M.J.V.C.); 2 Programa de Doctorado en Ciencias Morfológicas, Universidad de La Frontera, Temuco 4811230, Chile; danielconei@ug.uchile.cl; 3 Departamento de Ciencias de la Salud, Universidad de Aysén, Coyhaique 5951537, Chile; 4 Jefe Departamento Laboratorio Clínico, Hospital Clínico Universidad de Chile, Santiago 8380000, Chile

**Keywords:** SARS-CoV-2, COVID-19 vaccines, cell-mediated immunity, interferon-gamma release assays

## Abstract

In December 2019, a case of atypical pneumonia was reported in Wuhan, China. It was named COVID-19 and caused by SARS-CoV-2. In a few months, scientific groups around the world developed vaccines to reduce the disease’s severity. The objective was to evaluate the humoral and cellular immune response post immunization with three different vaccination schedules administered in Chile until January 2022. Sixty volunteers were recruited with a three-dose schedule, who had no history of infection nor close contact with a positive patient. IgG against the spike antigenic domain was detected, and the neutralization capacity against two groups of variants, Original/Alpha and Beta/Gamma, was also measured. Finally, the cellular response with interferon release was measured through IGRA. Results showed that there were significant differences in the neutralizing antibodies for the original and alpha variant when comparing three Comirnaty doses with Coronavac and Vaxzevria. A high number of reactive subjects against the different SARS-CoV-2 variants, alpha, gamma, and delta, were observed, with no significant differences between any of the three schemes, confirming the existence of a cellular immune response against SARS-CoV-2. In conclusion, the three vaccine schemes generated a cellular immune response in these volunteers.

## 1. Introduction

In December 2019, the scientific community was informed of an unidentified case of pneumonia in Wuhan, People’s Republic of China. This disease had similar characteristics to those that occurred in Guangdong Province, China in 2003 [1]. It is called coronavirus disease (COVID-19), which is caused by SARS-CoV-2, and during these two years of pandemic, it has killed more than five million people in the world. To prevent the transmission of SARS-CoV-2, restrictions on mobility and social interactions have been imposed on populations and during 2021, emphasis has been placed on mass vaccination of the world’s population [2,3].

SARS-CoV-2 is a positive single-stranded RNA virus with mantle and is covered by proteins with antigenic capacity such as spike (or protein S), which interact with its RBD domain with the cellular receptor ACE-2 to allow adsorption and subsequent viral replication in human cells [4,5,6]. Spike, in turn, is the molecule recognized by the immune system to prevent adsorption, that is, non-binding with the ACE-2 cell receptor, and therefore, it is a target for the design of COVID-19 vaccines, but also to adapt current platforms to mutations in this protein, particularly in the RBD domain [6,7]. T cells may provide protective immunity and limit serious disease in settings where antibody responses may be diminished [8]. In addition, T cells are capable of recognizing mutant variants of SARS-CoV-2 [9,10].

Two years after the beginning of the pandemic, scientists have learned how to recognize the virus’s morphology and pathophysiology. It was initially supposed to be a severe respiratory disease, like SARS-CoV-1. The initial respiratory manifestations, including severe pneumonia, were predominant, but extrapulmonary signs and symptoms were described during the next months like anosmia, ageusia, cough, and odynophagia. Clinical experience demonstrated that there are acute and subacute symptoms that may even prolong in time, many of them associated with the inflammatory response driven by cytokines, including cytokine storm [11].

In the COVID-19 acute phase, pain can be presented in diverse forms. One of the first reports showed that muscular pain affected up to 44% of the patients. There have been different types of pain described: myalgias/arthralgias, odynophagia, abdominal and thoracic pain, and headache [12]. 

Some authors have described a relation between IL-10 systemic levels and COVID-19, particularly pain related, showing a strong negative correlation between IL-10 systemic levels and pain intensity. IL-10 has been described through the years as an anti-inflammatory and analgesic cytokine. 

Not only has pain been related to COVID-19. A study conducted by the “Sex@COVID online survey” described the endothelial damage that was later associated with acute and long-term erectile dysfunction [13]. The authors suggest that this is a potential reason for mask use and vaccination [14].

Vaccination with a complete vaccination schedule is the most effective measure to prevent SARS-CoV-2 infection and to diminish the population’s viral load. As of 31 December 2021, the WHO has approved 10 vaccines for clinical use [15]. Vaccine use, and especially the complete scheme (two doses), has been associated with a decrease in COVID-19 severe cases, including hospitalization and deaths [16,17,18]. Vaccines not only prevent mortality but also decrease symptoms that may mean a repercussion in daily life worldwide. It has been demonstrated that they also help in reducing long-term COVID-19 effects [19], inferior respiratory infections, and erectile dysfunction associated with COVID-19 [13]. 

Chile has been one of the countries with the highest number of vaccinated per capita. Vaccination of the fourth dose has now begun and up to 24 January 2022, 16.9 million people have completed a vaccination schedule (two doses) [20], that is, 88.2% of the target population, (considered between 3 years of age and older) [21]. This country has had a new impetus in its national vaccination program in the wake of the pandemic, adjusting public policies to initiate Phase 2 and 3 studies for different vaccine models circulating in the world, including ChAdOx1 nCoV-19 now known as Vaxzevria [22,23], Coronavac [24], and Cansino [25]. This allowed the country to position itself as a safe and reliable place to carry out scientific studies on SARS-CoV-2, which included studies associated with the success of the third dose in the Chilean population [26], particularly in the increase of the humoral and cellular immune system response when using an inactivated vaccine as a triple dose compared to two doses of the same model [26,27,28].

Currently, scientists have planned new vaccine models in order to find vaccines that generate a greater cellular immune response and a longer duration of the immune response against SARS-CoV-2. In other words, in the new models, it has been proposed that the measurement of not only the humoral immune response, but also the cellular immune response, would allow a better assessment of a possible prolonged immunological memory [28].

Due to this background and in order to complement the information obtained in the literature, we have proposed the evaluation of the humoral and cellular immune response of T lymphocytes post-immunization with SARS-CoV-2 vaccines, according to the three vaccination schemes administered in Chile. This measurement was carried out after the inoculation of the third dose, according to current ministerial regulations, in a voluntary population assigned to the Hospital Clínico Universidad de Chile during January 2022.

## 2. Materials and Methods

Prospective observational study carried out at the Hospital Clínico Universidad de Chile in Santiago de Chile during January 2022.

We recruited 60 volunteer participants with complete primary vaccination schemes and boosters (three vaccine doses). They had not been infected with SARS-CoV-2 nor had close contact with a confirmed case. This was certified by the Chilean ministry database called EPIVIGILA which has the record of every test performed in the country in public and private health centers as well as all the demographic and clinical information of each person who suffered COVID-19 or was in close contact with a confirmed person. Additionally, they should have not consumed immunosuppressive or immunomodulatory medication. These patients were divided by the three different vaccination schemes authorized in the country. These were Sinovac pharmaceuticals known as Coronavac, Pfizer with the BNT162b2 vaccine, currently Comirnaty, and finally Oxford-Astrazeneca with Vaxzevria.

From the initial sample, 4 subjects inoculated with the Comirnaty-Comirnaty-Comirnaty scheme had cellular immune response triggered by nucleocapsid protein, protein which is not present in this type of vaccine, so they probably had controlled exposure to the virus due to the hospital service where they worked (Intensive Care Unit). Therefore, in order to ensure comparison between the groups, 4 subjects were excluded from the other vaccination schemes, which were selected according to the data’s dispersion based on confounding variables analysis. 

These participants, after signing an informed consent, were studied for both humoral and cellular immunity against the SARS-CoV-2 virus.

The participants were analyzed in 3 groups, those with primary and booster vaccination schedules.

- Coronavac-Coronavac-Vaxzevria (*n* = 16)
- Comirnaty-Comirnaty-Comirnaty (*n* = 16)
- Coronavac-Coronavac-Comirnaty (*n* = 16)

### 2.1. Evaluation of Humoral Response

Humoral response was measured in all patients using two techniques: (1) patient antibody titer measurements in the Vitros XT7600 equipment from Ortho Clinical diagnostics, and (2) an IgG antibody neutralization reaction measured by immunofluorescence in an SD Biosensor cassette.

The IgG Detection Immunoassay against the antigenic domain corresponding to the spike protein (S1) is a quantitative assay. The result is considered reactive and provides a numerical value in antibody binding units (BAU) starting at 17.8 BAU/mL, to its upper limit of measurement up to 200 BAU/mL (VITROS Immunodiagnostic Products Anti-SARS-CoV-2 IgG Quantitative Reagent Pack, Ortho-Clinical Diagnostics, Illkirch-Graffenstaden, France).

According to the manufacturer, this test has 94% (95% CI, 90.1–96.7%) sensitivity and 99.5% (95% CI, 99.3–99.99%) specificity [29]. 

Additionally, the detection of neutralizing antibodies was performed by a cassette neutralization reaction, which was measured by immunofluorescence, SARS-CoV-2 nAb FIA (Standard F Sd Biosensor, Suwon, Korea). This kit is intended for use as an aid in identifying individuals with an adaptive immune response to SARS-CoV-2. A subset of secreted antibodies that have been demonstrated in the laboratory to prevent SARS-CoV-2 viral entry to human cells are termed neutralizing antibodies. Neutralizing antibody tests for SARS-CoV-2 use purified proteins based on the key viral recognition, docking, and infection through the interaction of the SARS-CoV-2 spike protein binding domain (RBD) and ACE2 receptor. This kit was compared with WHO Reference panel materials and other kits in the market such as GenScript kit (virus neutralization test sVNT, Piscataway, NJ, USA). 

In this test, the result was interpreted as reactive over a 20 percent neutralization. According to the manufacturer, this test has 100% sensitivity and 99% specificity. This technique allows evaluating the neutralization capacity against two groups of variants, group 1 (FIA V1): Wuhan/Alpha (UK) and group 2 (FIA V2): Beta/Gamma Brazil/South Africa/Japan [30]. 

### 2.2. Assessment of the Specific Cellular Response of SARS-CoV-2

Cell-mediated immunity was assessed by measuring interferon gamma secreted by T cells in response to different antigens of the SARS-CoV-2 virus, using an IGRA kit specific for SARS-CoV-2 (interferon gamma release assay) detected by immunofluorescence (Covi-FERON FIA, SD Biosensor, Suwon, Korea).

IGRAs have been well studied and widely used in the field of tuberculosis diagnosis. In virology, this is their first time used. The production of interferon-gamma is a critical step in immunological defense mechanisms against SARS-CoV-2. 

Whole blood tubes were used as samples. In each kit’s tube, 1 mL of blood was injected. A total of 6 tubes per participant were used (nil tube, original protein S tube, variant protein S tube, Delta variant protein S tube, nucleocapsid protein tube, and mitogen tube), as specified by the manufacturer [31].

The kit includes the following tubes:

- Nil: patient’s baseline gamma interferon measurement
- Original SP: Tube with spike antigen of ancestral virus (Wuhan/Hu-1/2019) and UK/alpha lineage B.1.1.7 variant.
- Variant SP: Tube with spike antigen South African variant/Beta lineage B.1.351 and Brazilian variant/gamma lineage P.1.
- Variant S Delta: Tube with spike antigen variant Delta lineage B.1.617.2.
- NP antigen: tube with antigen corresponding to the nucleocapsid protein.
- Mitogen: positive control tube to rule out lymphocytic anergy.

After collecting the sample, it was incubated from 16 to 24 h at 37 °C and then centrifuged for 15 min at 2200–2300× *g*.

IFN-gamma was detected by the F2400 equipment by means of a fluorescent signal, delivering results in concentration (IU/mL) through the integrated software of the equipment (SD Biosensor).

For the subsequent interpretation of the results, the “cut-off value charts for STANDARD F Covi-FERON FIA” provided by the manufacturer were used.

According to the instructions from the provider, the interferon gamma value obtained by the patient’s tube was used and the result obtained in the Nil tube (patient’s baseline) was subtracted.

### 2.3. Statistical Analysis

For the statistical analysis, a descriptive analysis of the variables was carried out, expressing the quantitative variables as mean ± standard deviation and the qualitative variables as total sample size and total percentage (%). An analysis of confounding variables was performed, which included age, comorbidities (hypertension, type 2 diabetes mellitus, and obesity), and habits (smoking). Subsequently, antibody levels (Wuhan, Brazil, U.K. and South African in a quantitative way were analyzed), the reactivity of cellular immunity in a qualitative way, levels of interferon (IFN), percentage of neutralization of IgG anti-spike V1 FIA and V2 FIA, and titers of total and diluted CVT2QN antibodies in a quantitative way. Finally, a qualitative analysis of antibody titers according to reactivity was made.

For data processing, the GraphPad Prism 9.3.1 pro× (GraphPad Software Inc., San Diego, CA, USA, 2021) was used. Shapiro–Wilk was used as a data normality test. Depending on the normality of the data, the one-way ANOVA test with Tukey’s post-hoc test or the Kruskal–Wallis test with Dunn’s multiple comparisons test was used for quantitative variables. For qualitative variables, Chi square was used. A simple linear regression analysis was performed to calculate 95% confidence intervals in interferon levels according to the Wuhan variant, compared to the other variants; and in the percentage of neutralization of IgG anti-spike V1 FIA and V2 FIA. For the correlation analysis, the Pearson or Spearman correlation test was used, depending on the normality of the data, determining an *r* value. A *p* value < or = 0.05 was considered statistically significant (Table S1).

### 2.4. Ethical Considerations and Disclosures

All patients gave their written informed consent to participate in this study, which was approved by the ethics committee of the Hospital Clínico Universidad de Chile (Act of approval No. 66, Exempt Resolution No. 1014, Certificate OAIC 1237/22) this study was conducted following the guidelines of the Declaration of Helsinki and following the standards of good clinical practice of the World Health Organization (WHO).

## 3. Results

**Figure 1:**
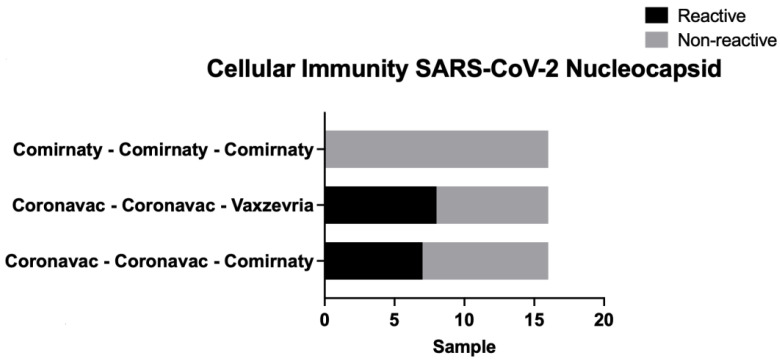
Comparison of cellular immunity against nucleocapsid (against NP) in subjects studied with the three vaccine dosage models at the HCUCH, January 2022.

There were no significant differences in the population in its general variables, except for age in those vaccinated with Vaxzevria booster dose (Table 1). In the comparison of the cellular immune response, there were statistically significant differences in the nucleocapsid protein tube variable, being lower in the group with the triple Comirnaty scheme (Table 2). When interpreted as a percentage, this also showed differences in the nucleocapsid for the same triple Comirnaty group (Table 3). The above is related to the number of subjects with this scheme, which were non-reactive from a qualitative point of view. Meanwhile, the mixed schemes had more reactive subjects (eight with Coronavac (two doses) + Vaxzeria scheme and seven with Coronavac (two doses) + Comirnaty scheme) (Figure 1).

**Figure 2:**
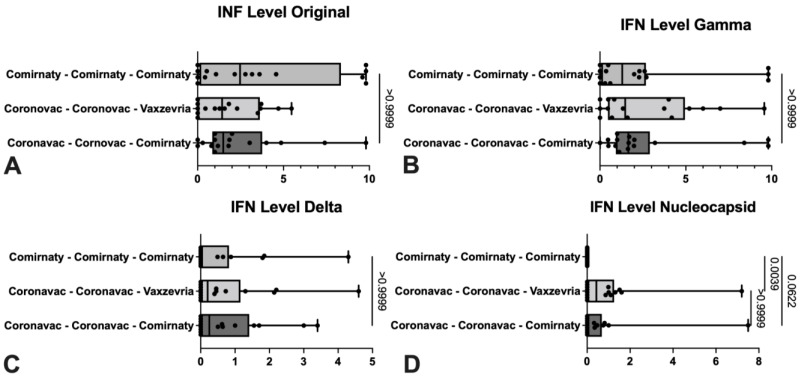
Comparison of cellular immune response through IGRA between the three vaccination schemes in subjects studied at HCUCH in January 2022.

**Figure 3:**
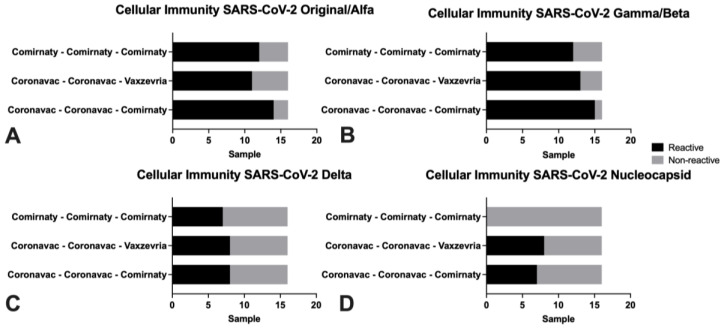
Interpretation of cellular immunity response when comparing IGRA for the three vaccination schemes in study subjects at HCUCH, January 2022.

Regarding interferon levels (IFN), there were significant differences in the nucleocapsid’s IFN level measure, between the groups with triple Cominarty scheme and mixed schemes (Coronavac complete scheme with Vaxzevria or Comirnaty booster doses) (Figure 2). Comparing the neutralization’s percentage, the group that presented the lowest values was the one immunized with Vaxzevria booster, with significant differences with the other vaccination schemes (Figure 3).

**Figure 4:**
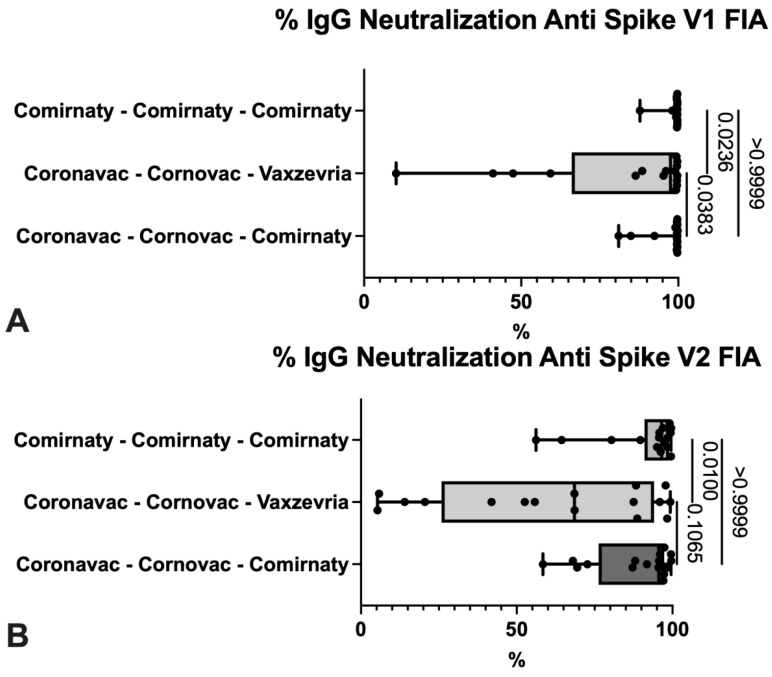
Comparison of the percentage of neutralization for two variants of spike protein according to the vaccine protocol under study. Subjects studied HCUCH, January 2022.

Regarding the CVT2QN titles, quantitatively, this group also presented the lowest average, with significant differences from the other two schemes (Figure 4).

In the linear regression analysis, when comparing the data with the IFN levels in the original variant, the lowest value was presented by the Delta variant in the triple Comirnaty scheme with *(p* = 0.0060; *r* = 0.6535; CI 95% [0.2334–0.8900]) (Table 4). When comparing the neutralization percentage of V1 FIA with V2 FIA, both mixed Coronavac schemes presented similar values (Table 5).

## 4. Discussion

In Chile, different vaccination schemes were used according to existing agreements with the pharmaceutical industry, largely due to phase 2 and 3 studies carried out at the beginning of vaccine production. Due to this, the entire Chilean population has had access to vaccines since December 2020. According to the authorizations issued by the Chilean public health institute (ISP) and European community [32,33,34], certain restrictions were considered for its application such as the use of the Astrazeneca laboratory vaccine (Vaxzevria) for people older than 55 years. Because of these restrictions, there was a significant difference observed in the age of the subjects recruited for the study of cellular and humoral immunity that we carried out at the HCUCH. The rest of the pathologies evaluated when recruiting the subjects are comparable between groups, such as tobacco, type 2 diabetes, hypertension, and obesity.

We found four subjects in triple Comirnaty scheme who had a cellular immune response to nucleocapsid protein, which is a finding that made us modify the study subgroups, because of the unfeasible result due to the vaccination model administered (mRNA vaccines, which only synthesize spike protein).

We therefore decided to eliminate these four subjects from the sample who were probably asymptomatic in their hospital functions prior to the study in question.

This work has the advantage of being the first in South America to compare cellular immunity in three different vaccine schemes in a population that has used three doses of SARS-CoV-2 vaccines. Therefore, it was interesting to observe the response to each of the schemes.

In Table 1 and Figure 2 and Figure 3, it is observed that only those vaccinated with Corona vac have a cellular immune response triggered by nucleocapsid antigenic protein. This is because of the vaccine’s type administered, where Coronavac vaccine is a viral inactivated vaccine of the SARS-CoV-2 virus, unlike the other platforms that use only the spike protein fragment to generate an immune response. There are studies in which the cellular immune response has been evaluated in some specific populations like patients with comorbidities, such as solid and hematological cancer and transplants, without considering multiple vaccination schemes in these studies [35,36,37].

A high number of reactive subjects can be observed against the different SARS-CoV-2 variants, both ancestral, alpha, beta, gamma, and delta, with no significant differences between any of the three schemes.

When performing the analysis of the humoral response, already known by the previous studies carried out in Chile and in the world [38,39,40], this particular study has the advantage of identifying neutralizing antibodies against some SARS-CoV-2 variants. It was observed that, with a complete vaccination schedule with a booster dose, there are high titers of antibodies and within these, neutralizing type antibodies. This has not been widely studied in world literature, let alone in South America [40,41,42].

In our investigation, it was observed that there were significant differences for the neutralizing antibodies for the original and alpha variant when comparing triple Comirnaty with Coronavac (two doses) + Vaxzevria and the same when comparing the latter with Coronavac (two doses) + Comirnaty. A lower percentage of neutralization was observed in the Coronavac (two doses) + Vaxzevria user group (Table 6).

According to the demographic data obtained, the only difference between the populations was their age, which could partially explain this difference in neutralizing antibodies, requiring further study to investigate other causes attributable to this difference. Similar results were obtained when looking for neutralizing antibodies for beta and gamma variants (FIA V2).

**Figure 5:**
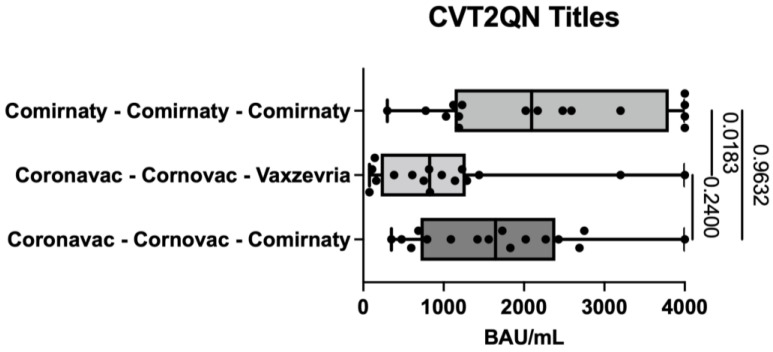
Measurement of antibody titers for the three vaccination schedules in study subjects at HCUCH, January 2022.

Regarding the measurement of antibody titers (Figure 5), this was done with an automated chemiluminescent immunometric technique with linear measurement up to 200 BAU/mL. Given the high titers of anti-spike protein antibody results, the team of specialists in the design of the technique was consulted, who recommended a dilution of 1/20 to achieve measurement linearity up to 4000 BAU/mL.

Unlike other articles, which compared the humoral immune response between different vaccines and found differences between them, as described in the investigation of Dashdorj et al. [43,44], in our study, we did not find statistically significant differences between the three vaccine schemes. All three schedules demonstrated excellent humoral response 2 months after the administration of the third dose (booster) (Figure 2). However, significant differences were observed in the measurement of neutralizing IgG antibodies against SARS-CoV-2 for the original and alpha variant (FIA V1) when compared to other variants, beta and gamma (FIA V2).

According to what was analyzed for the study of antibodies with the dilutions of the samples, it can be observed that all the vaccination schemes obtained high antibody titers, without great differences between groups. However, when performing the correlation analysis between the groups of vaccines with original variant and the other variants, such as beta and gamma, it was observed that there is a clear correlation for the Coronavac x2 + Comirnaty scheme and Coronavac x2 + Vaxzevria scheme, in which both have an r greater than 0, unlike triple Comirnaty, where there is no clear correlation, as shown in Table 5. This means, antibodies would only have a neutralizing effect for the original variant and not with delta in our group of subjects (*r* = 0.5902).

Within the limitations of our study, we highlight the low number of patients recruited per group with only 16 cases in each one, due to the limited access to IGRA and the recruitment time of the volunteers. However, we consider that this is pioneering work in South America that sets the start point on new studies in the cellular immune response against the COVID-19 pandemic.

To conclude, as a laboratory, we believe that the three schemes implemented by the Chilean government were useful in the prevention of SARS-CoV-2 from the perspective of the mechanism and functioning of RNA viruses from viral adsorption, neutralizing spike, preventing binding to ACE 2, defending through cellular immunity, and interferon effect. We believe that the next step will be the analysis of different vaccination scheme groups, evaluating the effect on the possibility of contagion and seeing its effect on the reduction of symptoms and the severity of the disease.

## 5. Conclusions

The vaccination schemes evaluated in this study showed a good humoral and cellular response, since there were no significant differences in antibody titers, neutralization percentage, or cellular immunity presence between the groups.

It is necessary to increase research on the cellular immune response as a complement to the humoral immunity obtained by SARS-CoV-2 vaccines. The new vaccines in development seek to enhance cellular immunity and studies like this show that this particular IGRA technique is a simple way to study this type of immunity in low and medium complexity laboratories from developing countries, as it is a friendly kit and easy to perform in any laboratory.

## Figures and Tables

**Table 1:** General background of the study population.

	Coronavac-Coronavac-Comirnaty	Coronavac-Coronavac-Vaxzevria	Comirnaty-Comirnaty-Comirnaty	*p* Value
*n*	16	16	16	-
Age ($\bar{x}$ ± *SD*)	35.31 ± 7.382	59.13 ± 3.828	35.94 ± 8.290	<0.0001 ^†^
Arterial Hypertension (%)	0 (0)	2 (12.5)	0 (0)	0.1241^‡^
Type 2 Diabetes Mellitus (%)	0 (0)	0 (0)	0 (0)	-
Obesity	0 (0)	2 (12.5)	0 (0)	0.1241 ^‡^
Smoking	0 (0)	2 (12.5)	0 (0)	0.1241 ^‡^

^†^ One-way ANOVA t and Tukey’s multiple comparisons. ^‡^ Chi-square test.

**Table 2:** Comparison of cellular immune response through IGRA between the three vaccination schedules in subjects studied at HCUCH in January 2022.

$\bar{x}\pm SD$	Coronavac-Coronavac-Comirnaty	Coronavac-Coronavac-Vaxzevria	Comirnaty-Comirnaty-Comirnaty	*p* Value
Nil Tube	0.1628 ± 0.0712	0.1794 ± 0.0940	0.1450 ± 0.0000	>0.9999 ^š^
Original SP Tube	3.236 ± 3.234	2.058 ± 1.876	3.206 ± 3.617	>0.9999 ^š^
Variant SP Tube	3.057 ± 3.320	3.003 ± 2.972	2.811 ± 3.611	>0.9999 ^š^
Spike Delta	0.9856 ± 1.132	0.9603 ± 1.280	0.7991 ± 1.167	>0.9999 ^š^
NP Tube	0.8503 ± 1.879	1.172 ± 1.783	0.1588 ± 0.0536	0.0078 ^š^
Mitogen Tube	10.000 ± 0.000	10.000 ± 0.000	10.000 ± 0.000	-

^š^ Kruskal–Wallis test and Dunn’s multiple comparisons.

**Table 3:** Interpretation of cellular immunity response when comparing IGRA for the three vaccination schedules in subjects studied at HCUCH in January 2022.

*n* ± %	Coronavac-Coronavac-Comirnaty	Coronavac-Coronavac-Vaxzevria	Comirnaty-Comirnaty-Comirnaty	*p* Value
Alpha/Beta	14 (87.5)	11 (68.75)	12 (75)	0.4380 ^‡^
Gamma	15 (93.75)	13 (81.25)	12 (75)	0.3499 ^‡^
Delta	8 (50)	8 (50)	7 (43.75)	0.9199 ^‡^
Nucleocapsid	7 (43.75)	8 (50)	0 (0)	0.0040 ^‡^

^‡^ Chi-squared test.

**Table 4:** Linear correlation of IFN levels measured by immunization schedule in original variant (Wuhan) in study subjects, HCUCH January 2022.

	Coronavac-Coronavac-Comirnaty			Coronavac-Coronavac-Vaxzevria			Comirnaty-Comirnaty-Comirnaty		
	Brazil	Delta	Nucleocapsid	Brazil	Delta	Nucleocapsid	Brazil	Delta	Nucleocapsid
*p* value	0.0055	0.0060	0.0023	0.0082	0.1097	0.2210	0.9265	0.8794	>0.9999
r	0.6591	0.6535	0.7056	0.6352	0.4153	0.3239	−0.02510	−0.04127	-
IC 95%	0.2427–0.8704	0.2334–0.8680	0.3229–0.8900	0.2037–0.8601	−0.1013–0.7555	−0.2046–0.7062	−0.5144–0.4765	−0.5262–0.4639	-

**Table 5:** Linear correlation of the neutralization percentage by immunization schedule for V1 FIA vs. V2 FIA, in study subjects at HCUCH, January 2022.

	Coronavac-Coronavac-Comirnaty	Coronavac-Coronavac-Vaxzevria	Comirnaty-Comirnaty-Comirnaty
*p* value	<0.0001	<0.0001	0.0161
r	0.8238	0.8618	0.5902
IC 95%	0.5545–0.9369	0.6392–0.9512	0.1336–0.8401

**Table 6:** Neutralization percentage of antibodies through IgG anti-spike measurement for variant V1 and V2 in the study’s population at HCUCH, January 2022.

$\bar{x}\pm SD$	Coronavac-Coronavac-Comirnaty	Coronavac-Coronavac-Vaxzevria	Comirnaty-Comirnaty-Comirnaty	*p* Value
V1FIA (ancestral/alfa)	97.08 ± 5.847	82.51 ± 27.60	98.78 ± 2.953	0.0236 ^š^
V2FIA (beta/gamma)	88.26 ± 13.38	61.80 ± 34.84	91.39 ± 13.13	0.0100 ^š^

^š^ Kruskal–Wallis test and Dunn’s multiple comparisons.

## Data Availability

Publicly available datasets were analyzed in this study.

## Supplementary Materials

The Database used in this investigation is available at https://www.mdpi.com/article/10.3390/life12040534/s1, Table S1: Cellular and Humoral immune response to different Chilean vaccines schemes Database.
